# Effect of different preservatives on the physicochemical characteristics and shelf stability of Rasmalai: A comparative study

**DOI:** 10.1002/fsn3.4019

**Published:** 2024-02-13

**Authors:** Tahir Mahmood Qureshi, Ghulam Mueen‐ud‐Din, Muhammad Nadeem, Ali Sirjan, Waseem Khalid, Salim‐Ur‐Rehman, Naushad Ahmad, Asad Nawaz, Muhammad Zubair Khalid, Felix Kwashie Madilo

**Affiliations:** ^1^ Department of Food Sciences Cholistan University of Veterinary and Animal Sciences Bahawalpur Pakistan; ^2^ Institute of Food Science and Nutrition University of Sargodha Sargodha Pakistan; ^3^ University Institute of Food Science and Technology The University of Lahore Lahore Pakistan; ^4^ Department of Food Science and Technology Riphah International University Faisalabad Pakistan; ^5^ Department of Chemistry, College of Science King Saud University Riyadh Saudi Arabia; ^6^ Institute for Advanced Study Shenzhen University Shenzhen Guangdong China; ^7^ Department of Food Science, Faculty of Life Sciences Government College University Faisalabad Punjab Pakistan; ^8^ Department of Food Science and Technology Ho Technical University Ho Ghana

**Keywords:** microbial counts, preservatives, Rasmalai, sensorial characteristics, shelf stability

## Abstract

Rasmalai is a very popular, delicious, and nutritious indigenous sweet dish in Indo‐Pakistani civilization. It has a very short shelf life, i.e., up to 3 days. The study was designed to assess the effect of preservatives (potassium sorbate and calcium propionate) on the shelf stability of Rasmalai. Moreover, proximate composition and sensory evaluation of prepared Rasmalai were also carried out in the present study. In general, potassium sorbate and calcium propionate significantly increased the shelf life of Rasmalai. But treatment (R_5_) containing a combination of both potassium sorbate and calcium propionate (500 ppm each) improved its shelf life by up to 12 days by keeping good sensorial characteristics. The maximum total plate counts as well as yeast and molds were observed in control Rasmalai (without any preservatives) whereas minimum counts were found in R_5_ treatment containing a combination of both potassium sorbate and calcium propionate (500 ppm each). In conclusion, all the preservatives used in the present study were effective in enhancing the shelf life of Rasmalai but R_5_ treatment containing a combination of both potassium sorbate and calcium propionate (500 ppm each) was the most effective in enhancing shelf life without deleterious effect on sensorial characteristics.

## Graphical abstract

All the preservatives used in the present study were effective in enhancing the shelf life of Rasmalai; however, both potassium sorbate and calcium propionate (500 ppm each) were the most effective in enhancing shelf life without deleterious effect on sensorial characteristics. Moreover, the maximum total plate counts as well as yeast and molds were observed in control Rasmalai (without any preservatives) whereas minimum counts were found in the combination of both potassium sorbate and calcium propionate (500 ppm each).

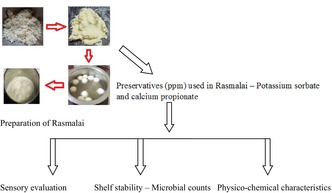

## 1 INTRODUCTION

Buffalo's milk is available in Pakistan for the manufacturing of different types of dairy products and sweetmeats. Along with the domestication of cattle, our ancestors of Indo‐Pakistani civilization adapted traditional ways to turn highly perishable milk into stable and sustainable dairy products. It is not surprising that Asians have a deep tradition of using milk and dairy products. The traditional dairy products of Indo‐Pakistani civilization may include fat‐rich products (ghee and butter), concentrated products (khoa and khoa‐based sweets and rabri), coagulated products (Rasogolla, Rasmalai, and paneer), fermented products (dahi and lassi), and frozen products (kulfi and kulfa).

Among the indigenous milk products, Rasmalai occupies special importance due to its delicious taste. It is a very popular chhana (paneer)‐based indigenous milk product in India, Bangladesh, and Pakistan and is usually consumed during summer season. Chhana or paneer is usually used as basic raw material for the preparation of Rasmalai. Different types of milk powders are available in the local market to prepare Rasmalai balls for the preparation of Rasmalai. People usually prepare Rasmalai by using milk powders but paneer is used in the original recipe of Rasmalai. Paneer balls and sweetened condensed milk are used for the preparation of Rasmalai (Sharma et al., 2015). It has been observed that Rasmalai available in the market starts to deteriorate after 3–4 days of storage in terms of its chemical, bacteriological, and sensory quality. The milk used for the preparation of Rasmalai may serve as an excellent culture medium for the growth of different types of microorganisms which ultimately influence its shelf stability. Obviously, limited shelf life of Rasmalai is a great deterrent to their commercial production. It is important for Rasmalai to reach the consumer in a usable condition. Different food preservatives can be used in preventing contamination of bacteria and mold during storage. The increase in shelf life of Rasmalai would be a nice idea for its higher consumption rate.

**TABLE 1:** Treatment plan of the present study.

Treatments	Preservatives (ppm) used in Rasmalai
R_0_	Control (Rasmalai) treatment (without addition of any preservative)
R_1_	Rasmalai prepared using 500 ppm potassium sorbate
R_2_	Rasmalai prepared by using 1000 ppm potassium sorbate
R_3_	Rasmalai prepared by using 500 ppm calcium propionate
R_4_	Rasmalai prepared by using 1000 ppm calcium propionate
R_5_	Rasmalai is prepared using a combination of both potassium sorbate and calcium propionate in equal quantity, i.e., 500 ppm each

To date, very scarce data are available regarding physicochemical characteristics and shelf stability of Rasmalai. The shelf stability and textural quality of Rasogolla have already been studied (Chakraborty & Bandyopadhyay, 2017; Das et al., 2010). Physicochemical characteristics of market available Rasmalai in India have also been studied (Sharma et al., 2015). To the best of our knowledge, no study has been carried out in Pakistan concerning shelf stability of Rasmalai by using selected preservatives. Keeping in view the shelf life of Rasmalai, current study was designed to monitor the effect of selected preservatives (potassium sorbate and calcium propionate) on physicochemical, microbiological, and sensory quality of Rasmalai.

## 2 MATERIALS AND METHODS

### 2.1 Procurement of raw material

Buffalo milk was procured from a local dairy farm in Sargodha city, Pakistan. Sugar, baking powder (NaHCO_3_), and soapnuts (locally known as Reetha) were purchased from the local market of Sargodha.

### 2.2 Preparation of paneer balls

Paneer was produced according to the method described by Khan et al. (2012) with some modifications. For all treatments, standardized milk (3% fat, 10 L) was used for the preparation of paneer balls as well as concentrated milk. Milk was pasteurized at 82°C for 5 min and then cooled to 72°C. At this stage, diluted lemon juice was added as coagulant with continuous but gentle stirring. The obtained coagulum was left undisturbed for approximately 10 min and the contents were allowed to cool up to 50°C. The whey was drained using a muslin cloth for 30 min by periodic squeezing of paneer matrix. The freshly prepared paneer was used as raw material for the manufacturing of Rasmalai balls which would be used as the main ingredient of Rasmalai.

### 2.3 Preparation of soapnuts (Reetha) extract

Five soapnuts were added to 100‐mL water and the extracts of soapnuts were prepared by heating at low flame. The extract was strained and cooled. The filtrate (extract) was ready to use to create foam in the sugar syrup while cooking paneer balls in it.

### 2.4 Preparation of Rasmalai and treatment plan

The preparation of Rasmalai was accomplished using traditional method to be used by our sweet manufacturers at local bakery plants. The ingredients used for the preparation of Rasmalai in the present study were different from those used by commercial bakeries. Commercial bakeries also use milk powder and corn flour as basic raw materials for making balls of Rasmalai. They also use semolina as well as small quantity of detergent (for creating foams in syrup). First of all, 50‐g corn flour and 7‐g baking powder were added into 1500‐g homogenous textured paneer matrix and kneaded until it became smooth. Small paneer balls (12–14 g) were prepared for making Rasmalai balls. A few spoons of extract of soapnuts were added into syrup of 55°Brix which created foams for even cooking of prepared paneer balls. Now, the prepared paneer balls were added into the pot containing sugar syrup and cooking was carried out at medium flame so that paneer balls were evenly cooked from all sides due to submerged condition. It was observed that the paneer balls took 10–12 min for proper cooking. When all the balls attained proper swelling without breakage, they were added into water after straining via sieve. About 8 L of raw buffalo milk (~6% fat) was also concentrated in a silver steel pot until the total volume became half. The sugar (1500 g) was then added into the concentrated milk and the mixture was heated on low flame until the brix reached 40°C. At this stage, the milk was known as concentrated milk having good sweet taste. Such type of concentrated milk usually resembles sweetened condensed milk (SCM). The paneer balls were added into concentrated milk after squeezing well so that no more water was left in the balls. The shelf stability of Rasmalai was studied by incorporating potassium sorbate and calcium propionate. Control (Rasmalai) treatment (without addition of any preservative) was designated as R_0_. The treatment denoted as R_1_ was the Rasmalai prepared using 500 ppm potassium sorbate whereas R_2_ was the treatment (Rasmalai) prepared by using 1000 ppm potassium sorbate. Rasmalai manufactured by using 500 ppm calcium propionate was denoted as R_3_ whereas R_4_ was the treatment (Rasmalai) prepared by using 1000 ppm calcium propionate. The last treatment (R_5_) was prepared using combination of both potassium sorbate and calcium propionate in equal quantity, i.e., 500 ppm each (Table 1). The mixture was placed in a refrigerator (4°C) for the whole night in order to make it ready to be consumed and analyzed.

### 2.5 Sampling of Rasmalai

All the samples from each treatment were put into plastic pot containing at least 200 g of Rasmalai and stored for 3, 6, 9, and 12 days (4°C). One fresh sample (without storage) was also analyzed. All the treatments (R_0_, R_1_, R_2_, R_3_, R_4_, and R_5_) were prepared in the same manner in three batches.

### 2.6 Physicochemical evaluation of prepared Rasmalai

For the analysis of Rasmalai, paneer balls and concentrated milk were blended into a homogeneous mixture. The pH of the mixture of Rasmalai was monitored using a pH meter (Ardö & Polychroniadou, n.d.). Acidity (%), moisture, and ash contents (%) of Rasmalai were determined by AOAC (AOAC International, 2012) method. The fat content of Rasmalai was determined by the Gerber–van Gulik method using a butyrometer. Total nitrogen (%) of Rasmalai was estimated according to the IDF standard 20B (IDF, 1993). Further, protein contents (%) of Rasmalai were calculated by multiplying total nitrogen (%) with 6.38.

### 2.7 Microbiological analysis of Rasmalai

The Rasmalai was analyzed for total plate counts (TPC, Log CFU/g) and yeast and mold counts (Y & M, Log CFU/g) during storage period by following the method described by Broadbent et al. (2013). Ten grams of homogenized Rasmalai sample was well mixed in 90 mL of sterilized tri‐sodium citrate (2%, pH 7.5) water. One milliliter of different dilutions of the above suspension (up to 10^−4^) was then plated on plate count agar media (Acumedia, LAB, Neogen® Culture Media, USA). The TPC were enumerated after incubating the petri plates for 2 days at 37°C. Similarly, the counts of Y & M were also done after 2 days at 30°C by employing potato dextrose agar (Biolife, Milano, Italia).

### 2.8 Sensory evaluation

The 9‐point Hedonic scale was used to evaluate the sensory attributes, i.e., appearance and color, flavor, sweetness, texture, as well as overall acceptability (Sharma et al., 2015) of prepared Rasmalai. A panel of 20 trained assessors was chosen including faculty and students from the Institute of Food Science and Nutrition, University of Sargodha, Pakistan.

### 2.9 Statistical analysis

The data were statistically analyzed using Statistix 8.1 software (Tallahassee, FL, USA). To compare the means, a two‐way analysis of variance (ANOVA) was used. Statistical significance was determined using a probability threshold of *p* < .05.

## 3 RESULTS AND DISCUSSION

### 3.1 Physicochemical characteristics of prepared Rasmalai

Table 2 presents the results of pH, titratable acidity (%), moisture (%), fat (%), protein (%), and ash (%) contents of Rasmalai investigated in the present study. There was significant (*p* < .05) effect of treatments and storage on the pH, titratable acidity (%), moisture (%), fat (%), protein (%), and ash (%) contents of Rasmalai. The pH of Rasmalai gradually decreased during subsequent storage periods. The pH values were more or less same as observed in all freshly prepared Rasmalai samples (6.52–6.66). The maximum decreasing trend of pH was observed in control samples (without preservatives) reaching 4.66 at the end of storage period (12 days). The lowest decreasing trend was observed in R_2_ and R_5_. Jouki et al. (2021) also observed decreasing trend of pH of SCM during storage. Similarly, Sharma et al. (2014) also reported decreasing trend of pH of concentrated part of Rasmalai. The decrease in pH during storage might be due to production of more and more acids. The production of acids might be due to the activities of contaminated microorganisms in Rasmalai during storage. In this way, they increased their number in Rasmalai mixture as it served as an excellent medium for their growth. On the other hand, the acidity of Rasmalai gradually increased during ripening. All the freshly prepared Rasmalai samples (treatments) showed lower contents of acidity. Among the treatments, R_0_ exhibited the maximum acidity (0.96%) after 12 days of storage while R_2_ showed the least value (0.45%) after 12 days of storage. The acidity of Rasmalai increased during storage due to the accumulation of more and more acids as a result of the activities of contaminated microorganisms.

**TABLE 2:** Physicochemical characteristics of Rasmalai during storage (0–12 days, 5°C) using different preservatives.

Treatments	Days	pH	Acidity (%)	Moisture (%)	Fat (%)	Protein (%)	Ash (%)
R_0_	0	6.62 ± 0.01 a–c	0.46 ± 0.01 k–m	47.56 ± 0.40 b	19.83 ± 0.29 e	11.73 ± 0.25 e	3.47 ± 0.02 e
	3	6.45 ± 0.02 f–h	0.55 ± 0.02 de	47.40 ± 0.35 c	20.02 ± 0.36 d	11.76 ± 0.12 e	3.51 ± 0.08 d
	6	5.88 ± 0.02 mn	0.67 ± 0.02 c	47.23 ± 0.45 d	20.10 ± 0.21 cd	11.91 ± 0.06 cd	3.66 ± 0.07 b
	9	5.22 ± 0.03 p	0.78 ± 0.01 b	47.07 ± 0.44 e	20.23 ± 0.06 c	12.05 ± 0.16 b	3.67 ± 0.02 ab
	12	4.96 ± 0.01 q	0.96 ± 0.01 a	46.93 ± 0.49 f	20.50 ± 0.35 a	12.25 ± 0.45 a	3.71 ± 0.05 a
R_1_	0	6.63 ± 0.01 a–c	0.44 ± 0.02 l–n	47.83 ± 0.76 ab	19.82 ± 0.15 e	11.64 ± 0.24 e	3.51 ± 0.02 d
	3	6.45 ± 0.02 f–h	0.47 ± 0.01 k–m	47.50 ± 0.23 b	19.96 ± 0.23 de	11.82 ± 0.23 d	3.55 ± 0.09 cd
	6	6.29 ± 0.02 k	0.52 ± 0.01 d–i	47.37 ± 0.36 c	20.16 ± 0.20 cd	11.98 ± 0.031 c	3.66 ± 0.05 ab
	9	6.12 ± 0.02 k	0.52 ± 0.02 d–g	47.06 ± 0.57 e	20.24 ± 0.10 c	12.08 ± 0.20 b	3.64 ± 0.0.06 b
	12	5.95 ± 0.05 lm	0.53 ± 0.01 d–f	46.76 ± 0.14 g	20.40 ± 0.40 b	12.13 ± 0.06 ab	3.75 ± 0.13 a
R_2_	0	6.66 ± 0.02 a	0.41 ± 0.02 n	47.98 ± 0.10 a	19.76 ± 0.15 e	11.71 ± 0.22 e	3.53 ± 0.53 d
	3	6.63 ± 0.01 a–c	0.44 ± 0.01 mn	47.73 ± 0.76 b	19.95 ± 0.53 de	11.88 ± 0.23 cd	3.63 ± 0.08 ab
	6	6.56 ± 0.01 c–e	0.44 ± 0.01 mn	47.46 ± 0.36 c	20.03 ± 0.17 d	11.89 ± 0.23 cd	3.66 ± 0.04 ab
	9	6.49 ± 0.02 e–h	0.44 ± 0.01 lmn	47.40 ± 0.64 c	20.12 ± 0.30 cd	12.07 ± 0.18 b	3.65 ± 0.05 ab
	12	6.43 ± 0.01 g–i	0.45 ± 0.01 lmn	47.05 ± 0.25 e	20.41 ± 0.20 b	12.21 ± 0.05 a	3.72 ± 0.10 a
R_3_	0	6.59 ± 0.01 a–c	0.45 ± 0.01 l–n	47.50 ± 0.02 a	19.73 ± 0.23 e	11.70 ± 0.26 e	3.50 ± 0.07 d
	3	6.42 ± 0.03 hi	0.46 ± 0.01 k–m	47.36 ± 0.76 c	19.83 ± 0.59 e	11.77 ± 0.07 e	3.51 ± 0.09 d
	6	6.13 ± 0.02 k	0.48 ± 0.01 h–m	47.13 ± 0.57 e	20.10 ± 0.55 cd	11.92 ± 0.07 cd	3.66 ± 0.06 ab
	9	5.98 ± 0.03 l	0.51 ± 0.01 e–k	46.96 ± 0.36 f	20.20 ± 0.23 c	12.07 ± 0.45 b	3.46 ± 0.07 e
	12	5.74 ± 0.04 o	0.66 ± 0.02 c	46.76 ± 0.26 g	20.43 ± 0.23 b	12.16 ± 0.00 ab	3.46 ± 0.12 e
R_4_	0	6.52 ± 0.03 d–f	0.48 ± 0.01 g–m	47.83 ± 0.76 a	19.71 ± 0.25 e	11.68 ± 0.14 e	3.52 ± 0.04 d
	3	6.35 ± 0.03 ij	0.50 ± 0.01 f–k	47.40 ± 0.32 c	19.86 ± 0.15 de	11.83 ± 0.22 cd	3.56 ± 0.07 cd
	6	6.08 ± 0.03 k	0.51 ± 0.02 d–j	47.27 ± 0.40 cd	20.03 ± 0.40 d	12.08 ± 0.25 b	3.60 ± 0.04 c
	9	5.96 ± 0.04 n	0.52 ± 0.02 d–h	47.06 ± 0.57 e	20.15 ± 0.21 cd	12.16 ± 0.22 ab	3.65 ± 0.03 ab
	12	5.86 ± 0.04 o	0.56 ± 0.01 d	46.90 ± 0.15 f	20.43 ± 0.26 b	12.36 ± 0.15 a	3.76 ± 0.05 a
R_5_	0	6.66 ± 0.02 ab	0.44 ± 0.01 l–n	48.02 ± 0.10 a	19.70 ± 0.30 e	11.89 ± 0.18 cd	3.56 ± 0.03 cd
	3	6.64 ± 0.02 ab	0.47 ± 0.02 i–m	47.83 ± 0.45 b	19.83 ± 0.26 de	11.82 ± 0.21 cd	3.51 ± 0.03 d
	6	6.61 ± 0.01 a–c	0.47 ± 0.01 j–m	47.53 ± 0.10 b	19.96 ± 0.15 de	11.94 ± 0.023 c	3.63 ± 0.05 bc
	9	6.56 ± 0.02 c–e	0.47 ± 0.01 i–m	47.22 ± 0.57 d	20.03 ± 0.12 d	12.05 ± 0.23 b	3.66 ± 0.08 ab
	12	6.50 ± 0.03 e–g	0.49 ± 0.01 f–l	46.76 ± 0.44 g	20.16 ± 0.22 cd	12.31 ± 0.05 a	3.72 ± 0.01 a

*Note*: Means with different letters in the same column show significant (*p <* .05) differences between treatments and storage period. R_0_ = Control (Rasmalai) treatment (without addition of any preservative); R_1_ = Rasmalai prepared using 500 ppm potassium sorbate; R_2_ = Rasmalai prepared by using 1000 ppm potassium sorbate; R_3_ = Rasmalai prepared by using 500 ppm calcium propionate; R_4_ = Rasmalai prepared by using 1000 ppm calcium propionate; R_5_ = Rasmalai prepared using combination of both potassium sorbate and calcium propionate in equal quantity, i.e., 500 ppm each.

The moisture contents (%) of Rasmalai slightly decreased during subsequent storage periods. The moisture contents of all freshly prepared Rasmalai samples were more or less same (47.50–48.02). The slight decreasing trend of moisture contents in Rasmalai might be due to some losses of moisture from the surfaces of Rasmalai mixture during storage period. Our results were concurrent to the findings of Sharma et al. (2014) who also observed slight decreasing trend of moisture contents during storage.

The protein contents (%) of Rasmalai slightly increased during subsequent storage periods. The protein contents of all freshly prepared Rasmalai samples were more or less same (11.64–11.89). The slight increase in protein contents might be due to a slight increase in dry matter of Rasmalai during storage. The fat contents (%) of Rasmalai also slightly increased during subsequent storage periods. The fat contents of all freshly prepared Rasmalai samples were more or less same (19.70–19.83). The slight increase in fat contents might be due to a slight increase in dry matter of Rasmalai during storage. Similarly, the ash contents (%) of Rasmalai slightly increased during subsequent storage periods. The ash contents of all freshly prepared Rasmalai samples were more or less same (3.47–3.56). The increase in ash contents during storage might be due to increased dry matter (due to decreased moisture contents) during storage period.

In addition, lactose as well as sugars would also be present in Rasmalai. The sugars also contribute to the dry matter of Rasmalai.

### 3.2 Total plate counts (TPC) and yeast and mold (Y & M) of Rasmalai

**FIGURE 1:**
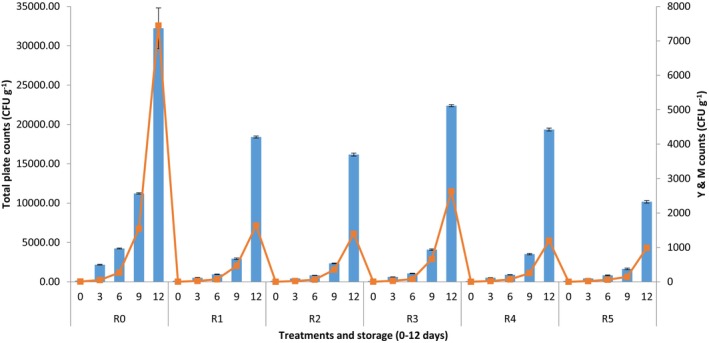
Total plate counts (TPC, means ± SD) (blue bars) and yeast and molds (Y & M, means ± SD) (red line) of Rasmalai during storage (0–12 days, 5°C) using different preservatives (R_0_ = Control (Rasmalai) treatment (without addition of any preservative); R_1_ = Rasmalai prepared using 500 ppm potassium sorbate; R_2_ = Rasmalai prepared by using 1000 ppm potassium sorbate; R_3_ = Rasmalai prepared by using 500 ppm calcium propionate; R_4_ = Rasmalai prepared by using 1000 ppm calcium propionate; R_5_ = Rasmalai prepared using combination of both potassium sorbate and calcium propionate in equal quantity, i.e., 500 ppm each).

Figure 1 shows the TPC and Y & M counts of Rasmalai samples prepared in the present study. There was significant (*p* < .05) effect of treatments and storage on TPC and Y & M counts of Rasmalai samples. All freshly prepared Rasmalai samples showed lower counts of TPC (86–153 CFU/g) but the counts increased during storage period. The maximum TPC counts (9233 CFU/g) were observed in control Rasmalai (R_0_) which was stored for 12 days whereas R_5_ showed the minimum counts (1316 CFU/g) at the end of investigated storage period (12 days).

Similarly, all freshly prepared Rasmalai samples showed lower counts of Y & M (1–7 CFU/g) but the counts increased during storage period. The maximum Y & M counts (1443 CFU/g) were observed in control Rasmalai (R_0_) which was stored for 12 days whereas R_5_ showed the minimum counts (386 CFU/g) at the end of investigated storage period (12 days).

The microorganisms may get entry into Rasmalai during manufacturing as well as postmanufacturing handling. The standard limit for the total counts in pasteurized milk should be up to 100,000 CFU/mL (GSO 1016:2015). Anderson et al. (2011) also observed standard plate counts in pasteurized milk up to 94,000 CFU/mL. The Rasmalai is basically a mixture of paneer balls and traditionally made concentrated milk from pasteurized milk (72°C, 20 s). In this way, paneer balls mimic paneer whereas concentrated milk resembles SCM. Renhe et al. (2017) observed the presence of mesophilic bacteria in SCM which may cause problems during storage. In another study, it was also observed that mesophilic and psychrotrophic microorganisms increased during storage in raw milk (Paludetti et al., 2018). These microorganisms can be expected in the concentrated milk which was prepared for dipping of paneer balls. It has already been investigated that bacterial as well as mold growth increased in paneer during storage (Qureshi et al., 2019, 2023). As milk provides an excellent medium for the growth of microorganisms due to the availability of plenty of nutrients and moisture contents, therefore the counts of microbes drastically increased during storage of such dairy‐based products. Even though the total plate counts as observed in our samples were within the standard limits, the mold growth was seen on the surfaces of Rasmalai samples (incorporated with preservatives, R_2_–R_5_) after an extended period of up to 15 days. Therefore, the samples stored for 15 days were discarded and hence not included for further analysis. The control samples started to deteriorate after 6 days of storage and they were considered unacceptable after 9 days of storage. Even though there was no visible mold growth in control samples stored for 6 days, slight change in flavor was observed at that stage. The control samples stored for 9 and 12 days were not acceptable due to visible mold growth.

There was a significant effect of preservatives in enhancing the shelf life of Rasmalai. Potassium sorbate (R_2_) and calcium propionate at the dose of 1000 ppm (R_4_) were effective in enhancing the shelf life of Rasmalai but the treatment having 500 ppm of each potassium sorbate and calcium propionate (R_5_) was the most effective in extending the shelf stability of Rasmalai. This means that the combination of preservatives would be a good strategy for enhancing shelf stability of Rasmalai.

### 3.3 Sensory evaluation of Rasmalai

**FIGURE 2:**
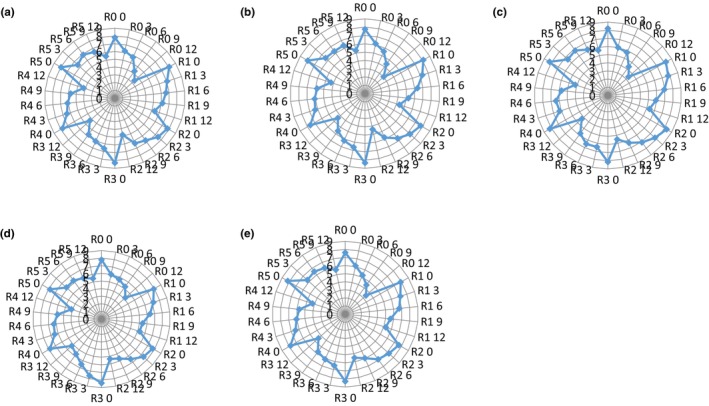
Sensory acceptance scores (means ±SD) regarding appearance and color (a), flavor (b), sweetness (c) body and texture (d), and overall acceptability (e) of Rasmalai during storage (0–12 days, 5°C) using different preservatives (R_0_ = Control (Rasmalai) treatment (without addition of any preservative); R_1_ = Rasmalai prepared using 500 ppm potassium sorbate; R_2_ = Rasmalai prepared by using 1000 ppm potassium sorbate; R_3_ = Rasmalai prepared by using 500 ppm calcium propionate; R_4_ = Rasmalai prepared by using 1000 ppm calcium propionate; R_5_ = Rasmalai prepared using combination of both potassium sorbate and calcium propionate in equal quantity, i.e., 500 ppm each).

Figure 2a–e presents the scores of sensory evaluation of Rasmalai prepared in the present study. It was observed that preservatives had significant effect on the scores of different sensory attributes, i.e., appearance and color, flavor, sweetness, texture, and overall acceptability. The scores for all sensory attributes of Rasmalai prepared in the present study decreased during storage. Among all the treatments, treatment having both potassium sorbate and calcium propionate in equal quantity, i.e., 500 ppm each (R_5_) showed the highest scores for appearance and color (5.50), flavor (5.30), sweetness (5.65), texture (5.45), and overall acceptability (5.60) even after 12 days of storage. The control Rasmalai samples (without any preservative) after 6 days of storage were not liked by the assessors. The control Rasmalai samples only attained maximum score until 3 days of storage but afterwards, mold growth was tremendous which appeared on the surfaces after 9 days of storage. The Rasmalai samples having different preservatives with low and high concentrations showed varying scores for all sensory attributes. The assessors evaluated Rasmalai samples very critically and observed prolonged shelf life by the use of different preservatives. The use of preservatives in combination was proved to extend the maximum shelf life of Rasmalai without any taste change. In a study conducted on the shelf stability of rasgolla, it was observed that the shelf stability of rasgolla increased up to 24 days by using sorbic acid (Das et al., 2010). They observed improved organoleptic properties of rasgollas during storage. Rasgolla is basically cooked paneer balls in syrup and resembles paneer balls of Rasmalai. As Rasmalai is a mixture of paneer balls and concentrated milk, therefore the shelf life was lower than rasgolla. Moreover, rasgolla has lower contents of moisture than Rasmalai. Therefore, shelf life of rasgolla was higher than Rasmalai as observed in the present study. In addition, another study also reported that shelf life of paneer is usually 6 days without the use of any preservatives (Qureshi et al., 2019) but it was observed that its shelf stability was enhanced up to 20 days by the use of potassium sorbate (Qureshi et al., 2019).

It is our general observation that bakeries produce Rasmalai by using powdered milk but their origin remains usually unknown. The Rasmalai prepared by the use of such inferior quality powders shows hard texture in the middle and gives appearance of uncooked material of powdered balls. Such type of Rasmalai may create digestion problems and therefore should be avoided. Hence, the traditional method should be adopted to prepare Rasmalai because paneer is used for making balls which usually enhance taste and texture without showing any kind of digestion disorders. Moreover, the use of preservatives within permissible limits would also be good idea to make Rasmalai samples to be available for consumption for a longer period.

## 4 CONCLUSION

On the basis of findings in the present study, it may be concluded that different preservatives were effective in delaying the microbial spoilage of Rasmalai but the treatment (R_5_) containing combination of both potassium sorbate and calcium propionate (500 ppm each) was the most effective in extending the shelf life up to 12 days without deleterious effect on sensorial characteristics.

## AUTHOR CONTRIBUTIONS

**Felix Kwashie Madilo:** Resources (equal); validation (equal); visualization (equal); writing – review and editing (equal). **Tahir Mahmood Qureshi:** Conceptualization (equal); data curation (equal); funding acquisition (equal); writing – original draft (equal). **Ghulam Mueen‐ud‐Din:** Data curation (equal); formal analysis (equal); investigation (equal); validation (equal). **Muhammad Nadeem:** Funding acquisition (equal); investigation (equal); methodology (equal); supervision (equal). **Waseem Khalid:** Data curation (equal); methodology (equal); supervision (equal); writing – review and editing (equal). **Salim‐ur Rehman:** Conceptualization (equal); formal analysis (equal); validation (equal); writing – original draft (equal). **Naushad Ahmad:** Methodology (equal); resources (equal); validation (equal); visualization (equal). **Asad Nawaz:** Data curation (equal); formal analysis (equal); project administration (equal); supervision (equal); visualization (equal). **Muhammad Zubair Khalid:** Methodology (equal); supervision (equal); visualization (equal); writing – review and editing (equal). **Ali Sirjan:** Conceptualization (equal); funding acquisition (equal); methodology (equal); supervision (equal); writing – original draft (equal).

## FUNDING INFORMATION

The authors would like to thank the Researchers Supporting Project number (RSPD2024R668), King Saud University, Riyadh, Saudi Arabia.

## CONFLICT OF INTEREST STATEMENT

The authors declare no conflict of interest.

## Data Availability

Data are contained within the article.

## References

[fsn34019-bib-0001] Anderson, M. , Hinds, P. , Hurditt, S. , Miller, P. , McGrowder, D. , & Alexander‐Lindo, R. (2011). The microbial content of unexpired pasteurized milk from selected supermarkets in a developing country. Asian Pacific Journal of Tropical Biomedicine, 1(3), 205–211. 23569760 10.1016/S2221-1691(11)60028-2 PMC3609194

[fsn34019-bib-0002] AOAC International . (2012). Official methods of analysis of AOAC International (19th ed.). AOAC Int.

[fsn34019-bib-0003] Ardö, Y. , & Polychroniadou, A. Laboratory manual for chemical analysis of cheese . Office for official publications of the Eurpean Communities. (Retrieved 12/10/2023).

[fsn34019-bib-0201] Broadbent, J. R., Brighton, C., McMahon, D. J., Farkye, N. Y., Johnson, M. E., & Steele, J. L. (2013). Microbiology of Cheddar cheese made with different fat contents using a Lactococcus lactis single‐strain starter. Journal of Dairy Science, 96(7), 4212–4222. 23684037 10.3168/jds.2012-6443

[fsn34019-bib-0004] Chakraborty, C. , & Bandyopadhyay, K. (2017). Textural analysis of spongy Indian milk dessert (Rasogolla) fortified with potato powder. International Journal of Current Microbiology and Applied Sciences, 6, 2414–2420.

[fsn34019-bib-0005] Das, D. , Alauddin, M. , Rahman, M. A. , Nath, K. K. , & Rokonuzzaman, M. R. (2010). Effect of preservatives on extending the shelf‐life of Rasogolla. Chemical Engineering Research Bulletin, 14(1), 19–24.

[fsn34019-bib-0006] IDF . (1993). Milk. Determination of nitrogen content. Kjeldahl method; Standard 20B. Annex I: Modified procedure for milk products. International Dairy Federation.

[fsn34019-bib-0007] Jouki, M. , Jafari, S. , Jouki, A. , & Khazaei, N. (2021). Characterization of functional sweetened condensed milk formulated with flavoring and sugar substitute. Food Science & Nutrition, 9(9), 5119–5130. 34532021 10.1002/fsn3.2477 PMC8441384

[fsn34019-bib-0008] Khan, S. U. , Pal, M. A. , Malik, A. H. , & Sofi, A. H. (2012). Process optimization for paneer production from milk powder. International Journal of Food Nutrition and Safety, 2, 62–71.

[fsn34019-bib-0009] Paludetti, L. F. , Jordan, K. , Kelly, A. L. , & Gleeson, D. (2018). Evaluating the effect of storage conditions on milk microbiological quality and composition. Irish Journal of Agricultural and Food Research, 57, 52–62.

[fsn34019-bib-0010] Qureshi, T. M. , Amjad, A. , Nadeem, M. , Murtaza, M. A. , & Munir, M. (2019). Antioxidant potential of a soft cheese (paneer) supplemented with the extracts of date (*Phoenix dactylifera* L.) cultivars and its whey. Asian‐Australasian Journal of Animal Sciences, 32(10), 1591–1602. 31011003 10.5713/ajas.18.0750 PMC6718899

[fsn34019-bib-0011] Qureshi, T. M. , Nadeem, M. , Iftikhar, J. , Ibrahim, S. M. , Majeed, F. , & Sultan, M. (2023). Effect of traditional spices on the quality and antioxidant potential of paneer prepared from buffalo milk. Agriculture, 13(2), 491.

[fsn34019-bib-0012] Renhe, Í. R. T. , Pereira, D. B. C. , SÁ, J. F. O. D. , Santos, M. C. D. , Teodoro, V. A. M. , Magalhães, F. A. R. , Perrone, I. T. , & Silva, P. H. F. D. (2017). Characterization of physicochemical composition, microbiology, sensory evaluation and microscopical attributes of sweetened condensed milk. Food Science and Technology, 38, 293–298.

[fsn34019-bib-0013] Sharma, S. P. , Kapoor, C. M. , Bisnoi, S. , Rani, M. , Jairath, G. , & Khanna, S. (2015). Scale of production, compositional, physico‐chemical and sensorial attributes of market samples of Rasmalai available in Hisar city of Haryana. India. Asian Journal of Dairy and Food Research, 34(1), 18–22.

[fsn34019-bib-0014] Sharma, S. P. , Kapoor, C. M. , Khanna, S. , Rani, M. , Bishnoi, S. , & Ahlawat, S. S. (2014). Technological aspects of indigenous chhana based Rasmalai. Haryana Veterinary, 53(2), 124–126.

